# Association between self-efficacy and general health: a cross-sectional study of the nursing population

**DOI:** 10.1186/s12912-021-00568-5

**Published:** 2021-03-21

**Authors:** Sakineh Dadipoor, Azin Alavi, Mohtasham Ghaffari, Ali Safari-Moradabadi

**Affiliations:** 1 Student Research Committee, Faculty of Health, Hormozgan University of Medical Sciences, Bandar Abbas, Iran; 2 Fertility and Infertility Research Center, Hormozgan University of Medical Sciences, Bandar Abbas, Iran; 3 Environmental and Occupational Hazards Control Research Center, School of Public Health and Safety, Shahid Beheshti University of Medical Sciences, Tehran, Iran; 4 Student Research Committee, School of Public Health and Safety, Shahid Beheshti University of Medical Sciences, Tehran, Iran

**Keywords:** Self‐efficacy, General health, Nurse

## Abstract

**Background:**

The present research aimed to explore the association of self-efficacy and general health among nurses.

**Methods:**

This was a descriptive-analytical. A total of 470 nurses were selected through the stratified sampling method. To collect the required data, GSE-10 and GHQ-28 were used. Independent-sample T-test, Pearson correlation coefficient, Chi-squared test, and regression were also used to analyze the obtained data.

**Results:**

The results reveal a statistically significant correlation between general health and self-efficacy (t=-6.72, *p* < .001). Among general health parameters, social functioning has significantly predicted self-efficacy.

**Conclusions:**

As shown in the present findings, an acceptable level of self-efficacy can positively affect all aspects of nurses’ general health.

## Background

The type of job is a key social determiner of health, which can affect individuals’ physical and psychological health statuses [1]. Nursing, as a stressful profession, is associated with a high level of skeletal/muscular stress, and psychologically speaking, it requires a high level of consciousness to respond to patients’ and their families’ medical needs as well as their key questions[2, 3]. Moreover, the limited sources in nursing work environment, fatigue induced by long working shifts [4], less support by nurses with senior ranks, and organizational changes can add to the above-mentioned problems [2, 5]. Several studies performed in Iran showed that about 93 % of nurses are subjected to the job-related stress [6]. Barzideh et al. in their research observed that 64.7 % of nurses are suspected of threats to their general health [7]. Furthermore, Rahmani et al. explored the aspects of nurses’ general health (GH) and also found that 50 % of nurses had an average level of GH and 62.7 % had a high level of anxiety. Additionally, 40.7 % of them had a low level of social mal-functioning and 47.5 % had a low level of depression. Overall, only 37.2 % of nurses had an acceptable level of GH [8]. In some other studies, none of the included nurses were found with an acceptable level of GH and more than half of them had a moderate level of GH [4]. In a body of research, the effects of self-efficacy on maintaining a positive attitude, reducing job burnout, and intensifying positive feelings were reported to be positive [9, 10]. In a number of studies, it has been reported that SE can improve one’s self-confidence in providing services under the complicated circumstances [11, 12]. In this regard, Bandura stated that SE is among the main factors in explaining behaviors and activities and controlling human functioning in social life [13].

As a positive source of resistance, self-efficacy (SE) is subsumed under a cognitive appraisal process, which is necessary for stress management. In addition, it is concerned with one’s ability of efficient performance in demanding situations. According to this stress management capacity, a vast majority of studies showed that SE affects mental health and psychological problems [14]. Nurses are among people whose SE scores are always estimated as low and this can adversely affect their other personality and work-related factors [15]. In another study, Lee aimed to explore the effect of SE, effectiveness, and collective efficacy on the functioning of nurses and as a result showed that the perceived SE is a main factor involved in nurses’ functioning, which is positively correlated with nurses’ performance [16]. According to Bandura, a low level of SE plays a key role in depression, anxiety, stress, psychosis, and other emotional states. Therefore, it makes sense that stressful factors are threats for those people who feel less competent during doing their duties at work [17].

To the best of the researcher’s knowledge, enlightened by reviewing the related literatures in different databases no similar work was found with the purpose of exploring the correlation of SE and GH among nurses. The related literature explored GH state and its correlation with the qualities of life and job burn-out [4, 8, 18]. The correlation between SE and clinical functioning of nursing students [19], SE and social health [20], SE and job-related stress [21], and critical thinking skills and EF beliefs were studied [22]. It seems that a gap exists in the performed studies on the correlation between SE and GH among nurses. Thus, the present research aimed to fill the existing gap and explore the correlation between SE and GH among nurses working in hospitals affiliated with Shahid Beheshti University of Medical Sciences.

## Method

### Design and participants

The present descriptive correlational research was conducted in 2020 in Tehran, the Capital of Iran and in those hospitals affiliated with Shahid Beheshti University of Medical Sciences. To fulfill this, 495 nurses were selected through the stratified randomized sampling method using their personnel codes. Data collection was done in January-February 2020. The sample was selected from different sections of hospitals.

Firstly, a brief introduction on the purpose of research was given to the participants and oral consent was then obtained from them. Notably, the questionnaires were submitted to be filled out as self-reports.

The research proposal and procedural protocol were reviewed and approved by the Ethics Committee of Shahid Beheshti University of medical sciences (code#IR.SBMU.RETECH.REC.1397.901).

### Instrumentation

In this study, GHQ-28 was used to assess the GH of nurses. Nursing is one of the professions for which this questionnaire is used, in order to evaluate its factors with a high frequency[6, 23].

### Demographic questionnaire

Using this questionnaire, some information such as age, gender, educational level, marital status, work experience, and number of work shifts were collected.

### General Health Questionnaire (GHQ-28) [24]:

This instrument, which consists of 28 items, delves into GH. Moreover, it is made up of four sub-scales each with 7 items as follows: physical symptoms (items 1–7), anxiety and sleep disorder (items 8–14), Social function, SF (items 15–21), and depression symptoms (items 22–28). It is noteworthy that this questionnaire explores individuals’ health over the past months. Respondents would provide answers to multiple-choice questions. Scoring of each item is between 0 and 3 and the total score is ranged from 0 to 84. For each respondent, 5 scores are finally reported, one for GH and four others for the sub-scales. Accordingly, a higher score represents a low health state. Therefore, GH level at different cut-off points can be interpreted as high (score 0 to 21), acceptable (score 22–42), moderate (score 43–64), and low (score 65–84). In this study, the cutoff point 23 was used for the new classification (sensitivity of 71.3 %, specificity of 90.6 %, and overall classification error of 11.7 %). Scores below 23 were considered as healthy and those equal to or above 24 represented a health-related problem. This questionnaire has been translated into Farsi by Iranian researchers and its validity and reliability have also been confirmed by some researchers[25, 26].

### Generalized Self-efficacy Scale (GSE-10) [27]:

This instrument was firstly developed by Schwarzer and Jerusalem in 1979 and then revised in 1981. It consists of 10 items, which all measure the level of general SE. The scoring is on a four-level Likert scale ranged from 1 to 4. An overall score between 10 and 20 is interpreted as a low SE, between 21 and 30 is taken as a moderate one, and above 30 is interpreted as a high SE. notably, the reliability and validity of the questionnaire were already established. In the present research, the internal consistency of the questionnaire was also estimated through Cronbach’s alpha, which was found to be 0.78.

### Statistical analysis

The data were entered into SPSS (SPSS Inc., Chicago, IL, USA) for performing the required statistical analyses. Independent-sample T-test, Pearson Correlation Coefficient, and Chi-squared test were used in this regard.

The predictive value of the variables was tested via regression analysis. Normality of the data was also tested through Kolmogorov-Smirnov test and the significance level was set at < 0.05.

## Results

The response rate was 95 % (470 out of 495). The average age of the included participants was 29.91 ± 6.08 years old. 86.6 % of them held a bachelor’s degree and 47.2 % had less than 5 years of work experience. The mean SE, GH scores, and other sub-scales are indicated in Table 1. The results reveal that only 1.3 % of the subjects had a high GH and 98.7 % of them had at least one problem related to their health status (from this rate, 3.6 % had a mild, 87.5 % a moderate, and 9.4 % a high level of health).

**Table 1:** The mean scores of variables

Variable		Mean ± SD
Self-efficacy		30.05 ± 6.64
General health		56.04 ± 8.03
	Somatic Symptoms	14.29 ± 3.02
	Anxiety And Sleep Disorder	14.46 ± 3.90
	Social Function	8.17 ± 2.82
	Depression Symptoms	19.10 ± 3.09

The results show a significant correlation between GH (two levels of high and low) and SE (t=-6.72, *P* < .001).

As the results indicate, 54.4 % of the participants had a high level of SE. The correlation between demographic features and SE level (low, moderate, and high) is included in Table 2.

**Table 2:** Relationship between demographic variables with self-efficacy

Variable	Subgroup	Self-efficacy			X2	p
		Low(*n* = 35)	Moderate(*n* = 179)	High(*n* = 256)		
General health status	Normal	0	6(3.4)	0	9.88	0.007
	adverse	35(100)	173(96.6)	256(100)		
Age (y)	< 30	17(48.6)	134(74.9)	144(56.3)	35.360	< 0.001
	30–40	12(34.3)	45(25.1)	74(28.9)		
	> 40	6(17.1)	0	38(14.8)		
Work experience (y)	< 10	17(48.6)	139(77.7)	160(62.5)	20.836	< 0.001
	10–20	18(51.4)	40(22.3)	90(35.2)		
	> 20	0	0	6(2.3)		
Gender	Male	0	32(17.9)	0	55.823	< 0.001
	Female	35(100)	147(82.1)	256(100)		

The correlation between demographic features and GH state is indicated in Table 3. Moreover, Chi-square test revealed no statistically significant correlation between gender and GH level (*P *> .05).

**Table 3:** Relationship of demographic variables with general health

Variable	Subgroup	General health				X2	p
		Variable normal	Slight disorder	Moderate disorder	Extreme disorder		
Age (y)	< 30	6(100)	17(100)	250(62)	22(50)	16.9.6	0.010
	30–40	0	0	114(28.3)	17(38.6)		
	> 40	0	0	39(9.7)	5(11.4)		
Work experience (y)	< 10	0	17(100)	266(66)	33(75)	23.249	< 0.001
	10–20	6(100)	0	131(32.5)	11(25)		
	> 20	0	0	6(1.5)	0		
Gender	Male	0	0	26(6.5)	6(13.6)	4.994	0.172
	Female	6(100)	17(100)	377(93.5)	38(68.4)		

As the regression analysis revealed, out of GH parameters, SF managed to predict SE significantly (Table 4).

**Table 4:** Factors predicting the self-efficacy of subjects based on general health

	Unstandardized Coefficients	Standardized Coefficients	t	Sig.
	B	Beta		
Somatic Symptoms	− 0.105	− 0.048	-1.007	0.315
Anxiety And Sleep Disorder	0.363	0.213	3.724	0.000
Social Function	-1.133	− 0.482	-12.112	0.000
Depression Symptoms	0.103	0.048	1.040	0.299

## Discussions

The present research aimed to explore the correlation between SE and GH among nurses. The results reveal a positive significant correlation between SE and aspects of GH. These findings are consistent with the results reported by Arabian et al. and Razmi et al. in their studies [28, 29]. Correspondingly, in these studies, SE was found as a strong predictor of GH. In fact, self-efficacy acts as a cognitive mediator, which affects individuals’ physical and psychological health statuses [20]. Nurses with a low self-efficacy were unable to show all their capabilities at workplace and this can consequently lead to a negative attitude toward themselves and their job and can also reduce their interests in job and satisfaction with work, which finally reduce GH.

As shown in the present research, there was a negative correlation among SE, anxiety symptoms, and sleep disorders. In fact, it was indicated that nurses with low SE are suffering from a low SF. This finding can be explained by the fact that one’s perception of self-capability is a key factor needed when facing anxiety-provoking conditions [30]. In their research, Cheung et al. suggested raising the level of SE for those nurses with anxiety [31]. Thus, holding educational sessions, lowering anxiety and stress before the entrance of nursing students into the medical field, and continuing such courses can help in confronting the above-mentioned problems.

As shown in the present research, SE and depression were negatively correlated. Chang et al. in their research found SE as a protective factor to adjust depression symptoms among nurses [32]. Accordingly, this finding is consistent with that of a research by Manojloch et al. [33]. Moreover, Luthans et al. reported that those with higher levels of SE probably have a better ability to strike a balance between their work and personal needs. These individuals also suffer from less emotional problems [34]. Moreover, a lower SE can probably lead to some behaviors among nurses such as depression, keeping a distance from patients’ rooms, and isolating themselves under demanding and sensitive health-related circumstances due to a lack of confidence in self-capabilities related to managing affairs [32].

In the present research, SE and SF were shown to be correlated significantly and positively. Correspondingly, this shows that nurses with a positive SE have a high self-efficacy, as well. As individuals’ SF can significantly promote their health level, it was shown that it is also involved in the occurrence, distribution, and continuation of the disease [35]. In another study, 13–14 % of nurses had some problems associated with SF induced by the limited supportive sources and conflicts in the workplace [36]. This finding pointed out the fact that the surrounding environment can play a key role in creating happiness and a positive feeling on what is happening in the workplace or on the contrary, it can create a negative attitude toward the workplace. Thus, it can be concluded that those nurses with a positive SF perceive events in workplace less threatening and this factor can increase the level of adaptation, which can consequently improve GH. It seems that an appropriate level of SF can be known as a potent resistance which can effectively contribute to psychological well-being of an individual. It is suggested that, to improve nurses’ SF, a warm, friendly, cooperative, and respectful atmosphere should be created in the workplace.

## Implications

As nursing is an influential job on social health, nurses need a high level of health from all aspects. However, due to specific work conditions, nurses usually face physical and psychological problems. Therefore, certain interventional measures can be taken into account to reduce such problems. In this regard, a number of suggestions are made here as follows: (1) Offering regular training and practical courses to better manage stress, (2) establishing a psychological consultation center only for nurses to improve their health and reduce stress and anxiety, (3) encouraging higher level managers and supervisors to create a more friendly, supportive, and collaborative environment to support the nursing staff morally. In this respect, some studies revealed that the reduced support from head-nurses can be a stress and anxiety-provoking factor in nursing work environment (3), (4) Avoiding intensive and unstandardized work shifts or employing more nurses can help in lowering stress level. In a study by Halpin et al., the role of high work load was mentioned as well as its positive correlation with nurses’ stress [37]. Thus, we recommend that the responsible authorities pay more attention to the improvement of nurses’ health and reducing their work hours due to the high work pressure, by recruiting more staff. And (5) Strategies suggested in this research help to lower depression, improve recreational mechanisms for nurses, creating leisure activities as planned in outer space of hospitals, and allowing regular leaves of absence.

## Limitations

There were certain limitations in the present research. Firstly, the participants only represented a particular geographical area in the capital of Iran, which makes the generalizability of the results impossible. However, it was attempted to select a large sample from various backgrounds to compensate for this limitation to a great extent. The other limitation was the self-report nature of the data collection procedure, which can lower the precision and validity of data. The present researchers attempted to ensure the participants of the confidentiality of the information they provided, to reduce this bias as much as possible. The absence of any similar research in terms of the purpose and statistical population to compare the results was another limitation of the research. Thus, it was attempted to examine a body of the related studies with similar variables.

## Conclusions

The present findings showed that SE is correlated with all aspects of GH. Thus, it is suggested to conduct further interventional studies to improve the self-efficacy among nursing staff and reduce stress, anxiety, and other negative aspects of work conditions.

## Data Availability

The datasets used and analyzed during the current study are available from the corresponding author on reasonable request.

## Abbreviations

GH: General Health
SE: Self-Efficacy
GHQ: General Health Questionnaire
GSE: Generalized Self-Efficacy Scale
